# Gut Microbiota Influences Experimental Outcomes in Mouse Models of Colorectal Cancer

**DOI:** 10.3390/genes10110900

**Published:** 2019-11-07

**Authors:** Alyssa A. Leystra, Margie L. Clapper

**Affiliations:** Cancer Prevention and Control Program, Fox Chase Cancer Center, Philadelphia, PA 19111, USA; alyssa.leystra@fccc.edu

**Keywords:** colorectal cancer, mouse models, microbiota, antitumor immunity

## Abstract

Colorectal cancer (CRC) is a leading cause of cancer-related deaths worldwide. Mouse models are a valuable resource for use throughout the development and testing of new therapeutic strategies for CRC. Tumorigenesis and response to therapy in humans and mouse models alike are influenced by the microbial communities that colonize the gut. Differences in the composition of the gut microbiota can confound experimental findings and reduce the replicability and translatability of the resulting data. Despite this, the contribution of resident microbiota to preclinical tumor models is often underappreciated. This review does the following: (1) summarizes evidence that the gut microbiota influence CRC disease phenotypes; (2) outlines factors that can influence the composition of the gut microbiota; and (3) provides strategies that can be incorporated into the experimental design, to account for the influence of the microbiota on intestinal phenotypes in mouse models of CRC. Through careful experimental design and documentation, mouse models can continue to rapidly advance efforts to prevent and treat colon cancer.

## 1. Introduction

Colorectal cancer (CRC) remains the second leading cause of cancer-related deaths worldwide [1]. Although research advances during the past decade have led to some of the most exciting breakthroughs in cancer treatment, including immune checkpoint blockade, the majority of CRC cases fail to respond to these new therapies [2,3]. A critical need exists to develop new strategies for the early detection, prevention, and treatment of colorectal cancer, as well as to elucidate the basis for the ineffectiveness of existing therapies. Such studies rely heavily on preclinical in vivo models that recapitulate the biology of human disease.

Studies in both chemically induced and genetically engineered mouse models of CRC have enhanced our understanding of colon tumor initiation, progression, and response to therapy. Such models continue to play an essential role in assessing promising chemopreventive, chemotherapeutic, and immunomodulatory agents for their ability to impact tumor development. However, as in humans, interpretation of the resulting study data is often compromised by significant inter-individual variability in tumor development and response to therapy. This heterogeneity can exist within a single genetically defined strain of mice and is even observed among caged littermates maintained under identical environmental and dietary conditions [4]. Our ability to refine existing in vivo models to more accurately mimic human colon tumor biology is predicated on a more in-depth understanding of the factors that contribute to phenotypic variability and impact therapeutic response.

Tumorigenesis and response to therapy in humans and mice alike are influenced by the microenvironment in which the colon tumor arises. Complex interactions among the commensal microbiota and the tissue-resident immune cells within the colon provide a dynamic microenvironment that is well equipped to rapidly respond to stimuli. Perturbations in the microenvironment directly impact the homeostasis of the colonic epithelium and dictate propensity for disease. Despite mounting evidence for the critical role the resident microbiota play in influencing the frequency of tumor initiation, rate of progression, and response to therapy, there is an underappreciation for these factors when selecting and developing animal models of CRC.

**Figure 1:**
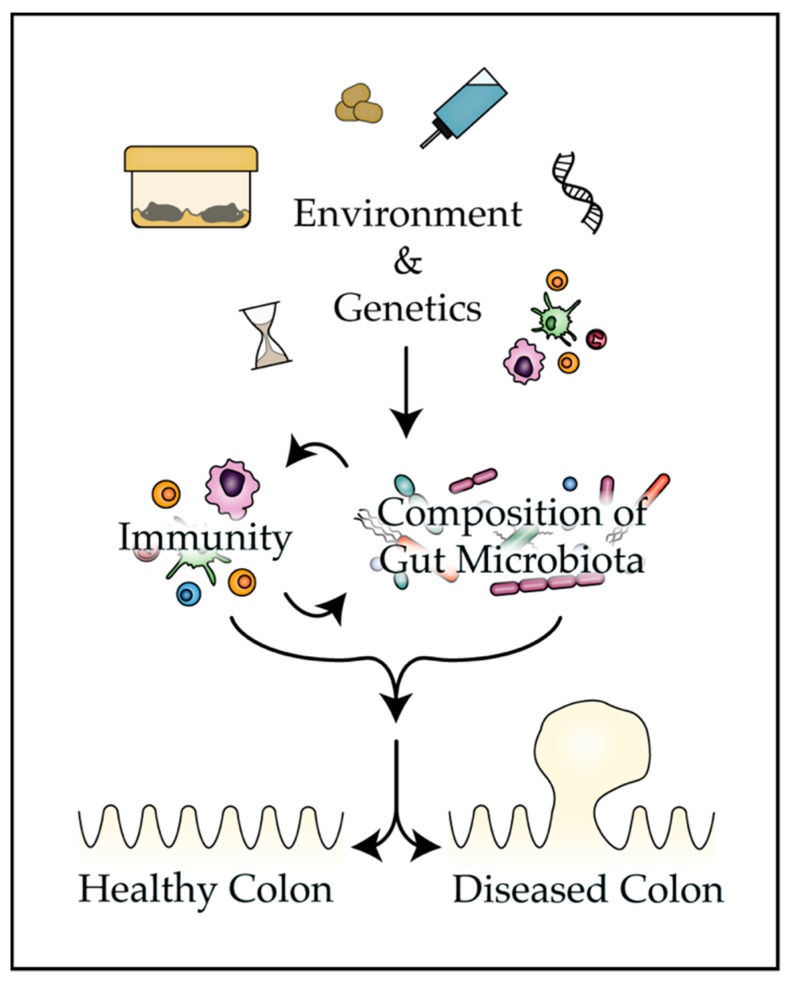
Many aspects of the murine environment can impact the composition of the gut microbiota, activity of the immune system, and ultimately the penetrance of disease phenotypes in mouse models of colon tumorigenesis.

The present review provides mounting evidence that the bacteria that colonize the mammalian gut play a pivotal role in tumorigenesis and the response to therapy in classic mouse models of CRC (Table 1). Numerous environmental and genetic factors are discussed that can impact disease phenotypes in mouse models by altering the composition of the gut microbiota (Figure 1). Finally, strategies are presented that investigators can employ to improve reproducibility and translatability of findings from mouse models of colon tumorigenesis and control factors that influence the composition of the microbiota.

## 2. Gut Microbiota Modulate Colon Tumorigenesis

The mammalian gut contains trillions of bacteria that coexist to form a complex ecosystem. Many of these microbes live in symbiosis with the host, metabolizing partially digested food, producing vitamins and nutrients, providing protection from opportunistic pathogens, and participating in the maturation, education, and activation of the tissue-resident and systemic immune system [21,22]. In delicate balance with the gut microbiota, the host epithelium and stroma form a tight barrier, consisting of a layer of mucin and antimicrobial products, in an attempt to protect the mucosa and underlying vasculature from invading microbes. Additional protection is afforded by the stroma of the gut, which is heavily infiltrated by tissue-resident immune cells that are poised to do the following: (1) respond rapidly to damage to the intestinal epithelium by producing wound-healing factors; and (2) mount a rapid response to invasion of the mucosal barrier by foreign microbes. The healthy colonic immune system is maintained as a balance of pro-inflammatory cells that are primed to respond to pathogens and danger-associated antigens and anti-inflammatory cells that suppress potentially damaging responses to commensal microbes and their byproducts. Perturbations in the composition of the gut microflora and/or direct contact of the microbes with the intestinal epithelium can skew the balance between pro-inflammatory and anti-inflammatory immune responses. Such alterations promote colon pathogenesis, leading to autoimmune activity, inflammatory bowel disease, and tumorigenesis.

Reduced microbial diversity, due to changes in the identity, richness, and relative abundance of microbial taxa, can be detected within the gut of mice with both spontaneous and chemically induced colon tumorigenesis prior to tumor formation. For example, *Apc*^Min/+^ mice spontaneously develop intestinal tumors due to a point mutation in codon 850 of the *Apc* tumor-suppressor gene. A reduction in microbial diversity was observed prior to the formation of visible tumors in C57BL/6J *Apc*^Min/+^ mice as compared to age and strain-matched C57BL/6J mice with wild-type *Apc* [23]. This decrease in diversity was driven primarily by an increase in the relative abundance of *Bacteroidetes* spp. within the colon of *Apc*^Min/+^ mice [23]. Similar findings have been reported for a murine model of chemically induced colitis-associated neoplasia. The azoxymethane/dextran sodium sulfate (AOM/DSS) mouse model is employed routinely as a prototypic model for the study of inflammatory signaling in a setting that recapitulates inflammation-associated colon tumorigenesis (ulcerative colitis) in humans. In this model, injection of the classic colon carcinogen AOM (single or multiple doses) initiates the colonic epithelium. Subsequent administration of DSS, a tumor-promoting agent, induces ulceration of the colonic mucosa followed by wound healing and proliferation of the epithelium. Interestingly, treatment with AOM/DSS resulted in broad shifts in the composition of the gut microbiota, as compared to that of healthy (untreated) mice, prior to the formation of visible tumors [15]. An increase in the relative abundance of members of the *Bacteroides* genus and a concomitant decrease in members of the *Prevotella* genus and unclassified genera within the Porphyromonadaceae family led to the observed reduction in diversity [15]. Taken together, these findings from spontaneous and chemically induced colon tumor models indicate that differences in the composition of the microbiota can be detected in mice that eventually develop tumors compared to mice that do not.

### 2.1. Evidence that Microbiota Can Restrain Colon Tumorigenesis

Depletion of microbiota can lead to an increase in the incidence and multiplicity of colon tumors in some mouse models of CRC. Intermittent, long-term administration of broad-spectrum antibiotics to *Apc*^Min/+^ mice produced shifts in the microbial composition of the gut and increased tumor number over time [5]. Exposure to antibiotics caused a dramatic decrease in the overall abundance and diversity of microbes, as indicated by an increase in the relative abundance of three genera (*Enterococcus*, *Ureaplasma*, and *Peptoclostridium*) and a decrease in many others (including *Bacteroides*, *Lactobacillus*, and *Desulfovibrio*). Antibiotic treatment caused a ~1.5-fold increase in the number of tumors throughout the intestine. Likewise, antibiotic use is associated with an increased risk of colon tumorigenesis in humans [24]. Thus, reduced microbial abundance may lead to an increase in the incidence and multiplicity of colon tumors in humans and mouse models alike.

Animals exhibiting a complete absence of commensal microbiota face an increased risk of developing colon tumors following exposure to an inflammatory stimulus. Germ-free (GF) AOM/DSS mice developed more tumors than conventional AOM/DSS mice that had been colonized with diverse and largely undefined microbiota since birth [16]. Delayed activation of tissue-repair pathways was observed within the gut of AOM/DSS-treated GF animals lacking commensal bacteria. Animals colonized with microbiota exhibited an acute inflammatory response to initial DSS treatment within 12 days, as characterized by increased cytokine signaling and recruitment of inflammatory cells involved in tissue repair. In contrast, GF animals failed to initiate an acute inflammatory response to the epithelial damage induced by DSS; tissue repair did not begin until 3–4 weeks later. The delayed onset of tissue repair in GF mice relative to conventional mice was coupled with dysregulation of repair pathways, resulting in hyperproliferation and formation of microadenomas. Ultimately, GF mice developed more and larger tumors than conventional mice. The increased tumor burden observed in GF mice was partially reversed by administering lipopolysaccharide, a microbial byproduct, in the drinking water. Complete rescue was achieved by colonizing GF mice with microbiota from conventional mice prior to AOM/DSS treatment. Together, these findings suggest that commensal microbes and their byproducts assist in the initiation of appropriate tissue repair and recovery from inflammatory insults.

Laboratory mice colonized with microbiota from wild-caught mice displayed a dramatic increase in gut microbial diversity and a decrease in the number and size of AOM/DSS-induced tumors relative to conventional AOM/DSS-treated laboratory mice [17]. Changes in the phylum-level composition of the gut microbiota included an increased relative abundance of Bacteroidetes and Proteobacteria and a reduction in Firmicutes, Tenericutes, and Verrucomicrobia in wild-caught vs. C57BL/6 mice. Following AOM/DSS treatment, C57BL/6 mice colonized with microbiota from wild-caught mice developed less inflammation and ~3-fold fewer colon tumors than AOM/DSS-treated C57BL/6 mice colonized with microbiota from conventional laboratory C57BL/6 mice. Thus, increased diversity of the gut microbiota was associated with decreased tumorigenesis in the AOM/DSS-treated C57BL/6 model, perhaps due to modulation of the inflammatory response to DSS treatment.

Colonization of mice with mixtures of specific strains of bacteria can reduce tumorigenesis. One such mixture of bacteria, a probiotic called “VSL#3”, consists of *Lactobacillus casei, Lactobacillus pantarum, Lactobacillus acidophilus, Lactobacillus delbrueckii* subsp. *Blugaricus, Bifidobacterium longum*, *Bifidobacterium breve*, *Bifidobacterium infantis*, and *Streptococcus salivarius*. Administration of VSL#3 to conventional mice resulted in a significant decrease in the number and size of colon tumors per mouse following AOM/DSS treatment [25,26]. These experiments provide proof-of-concept that modulation of the abundance of specific strains of bacteria in the gut can restrain colon tumorigenesis.

### 2.2. Evidence that Microbiota Can Promote Colon Tumorigenesis

Microbiota likely perform dual roles in tumorigenesis. Many commensal species protect against invasion of the gut epithelium by pathogens, while other species promote inflammation and pro-tumorigenic signaling. Thus, depletion of bacteria can be either harmful or beneficial in a context-dependent manner. In contrast to the above studies where microbial depletion resulted in increased tumorigenesis, other studies have yielded opposing data. Mice treated with broad-spectrum antibiotics prior to and throughout AOM/DSS administration developed ~2-fold fewer tumors than those that didn’t receive antibiotics [15]. Similarly, treatment of *Cdx2-Cre Apc*^flox/+^ mice with broad-spectrum antibiotics led to a 2-fold decrease in the number of spontaneous colon tumors, as compared to untreated animals [27]. Furthermore, GF and broad-spectrum antibiotic-treated *Apc*^Min/+^ *Msh2*^−/−^ mice with microsatellite instable disease developed fewer colon tumors than conventional mice [14]. When combined, these studies provide convincing evidence for the pro-tumorigenic role of microbiota in a number of distinct animal models.

Experiments involving the inoculation of mice with microbiota from either healthy or diseased hosts provide direct evidence of the ability of the microbiota to either protect against or promote tumorigenesis. Mice colonized with gut microbiota from tumor-bearing animals prior to treatment with AOM and DSS developed ~2-fold more colon tumors than similarly treated mice colonized with a ‘healthy’ gut microbiota obtained from tumor-free mice that were not treated with either agent [15]. Similarly, AOM-treated mice colonized with microbiota from CRC patients developed more severe inflammation as well as a higher tumor incidence and grade than mice that received microbiota from healthy donors or no microbiota [28]. These findings demonstrate that the composition of the gut microbiota can impact inflammatory responses and subsequent tumor formation in mouse models of colon tumorigenesis.

### 2.3. Contribution of Specific Bacteria to Colon Tumorigenesis

Clearly, the composition of the gut microbiota influences the incidence and multiplicity of colon tumors that develop in mouse models. However, only a few individual species of human microbes have been identified that reproducibly promote colon tumorigenesis in immunocompetent mouse models.

#### 2.3.1. *Fusobacterium Nucleatum*

*Fusobacterium nucleatum*, a commensal microbe that can become pathogenic under conditions of reduced microbial diversity, is enriched within the colon of patients with colonic adenomas and/or cancer as compared to healthy subjects [29]. *Apc*^Min/+^ mice colonized with *F. nucleatum* develop more aberrant crypt foci and tumors in the colon than sham-colonized *Apc*^Min/+^ mice, as well as a greater number of small intestinal adenomas and adenocarcinomas [29]. Tumors from mice colonized with *F. nucleatum* exhibit higher expression of pro-inflammatory genes, including *Ptgs2*, *Il1b*, *Il6*, *Il8*, *Tnf*, and *Mmp3*, than tumors from sham-colonized controls. These findings suggest that *F. nucleatum* may drive the development and progression of intestinal tumors via activation of inflammatory pathways.

#### 2.3.2. *Bacteroides Fragilis*

*Bacteroides fragilis*, another common commensal bacterium, has also been implicated in the development and growth of colon tumors in both humans and mice [6,7,8,9]. Enterotoxigenic *B. fragilis* (ETBF) induces colon tumorigenesis in *Apc*^Min/+^ mice by inducing pro-inflammatory IL-17 and NF-κB signaling throughout the colonic mucosa [6,8]. Prompt clearance of ETBF with the antibiotic cefoxitin can mitigate these effects [7]. Use of a nontoxigenic strain of *B. fragilis* that does not produce BFT upon colonization is insufficient to enhance either inflammatory signaling or tumorigenesis as compared to sham-colonized *Apc*^Min/+^ mice. Thus, toxin production by EBFT is required for induction of inflammatory signaling and subsequent enhanced tumorigenesis [6].

#### 2.3.3. *Escherichia Coli*

Strains of *Escherichia coli* that carry the *pks* gene locus and thus produce colibactin, a known genotoxin, enhance tumor formation and growth in both humans and mice. Expression of genes at the *pks* island of *pks+ E. coli* is enhanced in mice during carcinogenesis [30]. Colonization of GF *Il10*^−/−^ mice, which are susceptible to inflammation due to deletion of the *Il10* gene, with either *pks+* or *pks− E. coli* results in the induction of severe colitis [10]. Colonization of AOM-treated GF *Il10^−/−^* [10] or AOM/DSS [31] mice with *pks+ E. coli* increased the number of colon tumors per animal beyond that of mice colonized with *pks− E. coli*. In contrast, *pks+ E. coli* was insufficient to induce either colonic inflammation or tumorigenesis in AOM-treated GF *Il10^+/+^* (wild-type) mice [10]. Together, these findings indicate that expression of the *pks* locus in *E. coli* may cooperate with inflammation to promote tumor growth and progression within the colon.

### 2.4. Activities of the Microbiota that Impact the Homeostasis of the Colonic Mucosa

#### 2.4.1. Biofilm Formation

Under homeostatic conditions, the colonic epithelium produces a layer of mucin that serves as a barrier between the commensal microbiota and the colonic mucosa. Successful invasion of the mucin layer by bacteria results in the formation of a dense, matrix-enclosed aggregation of multiple species of bacteria that adhere tightly to surfaces [32]. Such biofilms perform a potentially pathogenic function within the colon by doing the following: (1) bringing the bacteria in closer proximity with the mucosal surface of the colon; (2) protecting the bacteria from external insults, including antibiotic treatment; and (3) facilitating cooperation between multiple bacterial species through nutrient exchange and horizontal gene transfer, to enhance survival of the community [32]. In humans, bacterial biofilms are frequently detected on adenomas and cancers alike; one study identified biofilms on 50% of the CRCs examined [33]. These biofilms are not restricted to the tumor, but can extend to the nontumor tissue. Normal tissue covered with biofilm displays pro-tumorigenic changes, as depicted by reduced crypt cell expression of E-cadherin, increased epithelial cell expression of IL-6, and increased proliferation relative to biofilm-free tissue [33]. These changes, which occur irrespective of whether the tissue is from a CRC patient or healthy subject, provide evidence that the biofilm may promote tumorigenesis. 

Biofilms found in the colons of CRC patients are commonly co-colonized with *E. coli* and ETBF [9]. Co-colonization of *Apc*^MinΔ716/+^ mice or AOM-treated wild-type mice with pathogenic strains of both *pks+ E. coli* and ETBF significantly increased colon tumor multiplicity relative to co-colonization with a single pathogenic strain of either *E. coli* or ETBF and the nonpathogenic strain of the other. Co-colonization with both pathogenic strains of bacteria also resulted in enhanced *pks+ E. coli* invasion into the biofilm and tissue relative to colonization with *pks+ E. coli* alone. This observation suggests that ETBF may be required to break down the protective mucus layer, thus allowing *pks+ E. coli* to come in direct contact with epithelial cells. Thus, multiple species of pathogenic bacteria can cooperate within biofilms to promote tumorigenesis.

While colonic biofilms containing pathogenic bacteria promote tumorigenesis, biofilms colonized by beneficial bacteria could hypothetically promote host defense and protect against tumorigenesis. Bacterial biofilms can promote good health in some tissues, such as the oral cavity [34]. However, the existence of similar health-promoting roles for intestinal biofilms remains controversial [32]. Additional research is required to determine if biofilms containing nonpathogenic bacteria can form and persist in close contact with the healthy colonic mucosa and, if so, what impact (if any) they have on colon cancer risk and treatment response.

#### 2.4.2. Metabolites Produced by Microbiota 

Intestinal bacteria participate in the metabolism of carbohydrates, lipids, and amino acids that pass through the gut. Products of this metabolism influence the pH, oxidative environment, energy availability, and presence of carcinogens in the microenvironment of the colonic mucosa [35,36]. Many of these metabolites either promote or restrain colon tumorigenesis. For example, the genotoxin colibactin, produced by *pks+ E. coli*, promotes tumorigenesis by inducing DNA damage [37]. In contrast, colonization of GF mice with *Butyrivibrio fibrisolvens* increases the amount of the protective short chain fatty acid (SCFA) butyrate within the colon relative to uncolonized GF mice [38]. Butyrate production is inversely correlated with tumor multiplicity in AOM/DSS-treated mice; *B. fibrisolvens*-colonized mice fed a high fiber diet develop 3-fold fewer colon tumors than GF mice maintained on the same diet. This protective effect is lost when mice are colonized with a mutated strain of *B. fibrisolvens* incapable of metabolizing soluble fiber to butyrate, providing strong evidence for the ability of butyrate to inhibit tumor formation in these animals. Butyrate and other SCFAs likely modulate colon tumor formation in part by reducing colonic inflammation [39,40]. Mice deficient in SCFA receptors exhibit more severe colitis in response to repeated DSS treatment relative to wild-type mice [39]. In addition, SCFA receptor-deficient animals develop greater numbers of colonic tumors after AOM/DSS treatment than wild-type mice. Interestingly, SCFAs can induce the activation and expansion of colon-resident immune cells and attenuate colonic inflammation in a T-cell transfer model of colitis [40]. The anti-inflammatory properties of butyrate cooperate with other chemopreventive dietary components including (*n*-3) polyunsaturated fatty acids (PUFAs) to dramatically reduce severity of inflammation, accumulation of DNA damage, risk of tumor formation, and growth of tumor cells [41]. These findings illustrate that production of metabolites by gut microbial communities may promote or restrain colon tumorigenesis by modulating DNA damage and inflammatory immune responses to harmful stimuli. Thus, evaluating metabolite production by the gut microbiota may provide greater mechanistic insight into how microbiota interact with the colonic mucosa to either promote or restrain colon tumorigenesis.

#### 2.4.3. Interactions with Tissue-Resident Immune Cells

Gut microbiota shape the maturation of the immune system and its activation in mice, starting at a young age. Mice that remain GF throughout development exhibit abnormally organized immune organs, including spleens and lymph nodes, as compared to pups that are colonized with microbes at birth [42]. Although many of these defects can be corrected by microbial colonization later in life, some persist [43]. For example, mice that do not encounter bacterial antigens prior to weaning exhibit immune intolerance to bacterial antigens upon gut-barrier disruption by DSS in early adulthood (30 days of age), resulting in increased expansion of gut mucosal T cells, fewer Tregs, and more severe colitis than mice exposed to bacterial antigens prior to weaning [44]. 

Gut microbiota regulate the recruitment and activation of pro-inflammatory lymphocytes, including T-helper 17 (Th17) and γδ T-cells within the colon [45,46]. For example, inoculation of germ-free mice with feces from patients with CRC resulted in increased recruitment of Th17 cells to the colon as compared to mice inoculated with feces from healthy subjects [28]. Invasion of the colonic epithelial layer by gut microbiota also enhanced Th17 cell recruitment and stimulation [27,47,48]. Similarly, mice colonized with bacteria exhibited greater γδ T-cell recruitment and stimulation in the intestine than antibiotic-treated or GF mice [46]. These lymphocytes in turn produced pro-inflammatory cytokines, including IL-23 and downstream IL-17, upon activation. IL-17 production from tumor-infiltrating Th17 and γδT cells increased colon tumor development in *Apc*^Min/+^ mice [49]. Conversely, ablation of IL-23 signaling in immune cells resulted in reduced expression of IL-17 and fewer tumors within the colon of *Cdx2-Cre Apc*^flox/+^ mice [27]. Together, these findings indicate that pro-inflammatory signaling by the adaptive immune system can promote colon tumor growth. 

Gut microbiota also regulate the recruitment and activation of the immunosuppressive T regulatory cell (Treg) population within the colon. Tregs are common residents of the colon and oppose the activity of pro-inflammatory lymphocytes. Colons of GF mice harbor fewer Tregs than colons of conventional mice, a phenotype which could be rescued by colonization of mice with strains of *Clostridium* but not by colonizing with other bacterial strains [50]. Colonization of GF mice with a mixture of 17 strains of Clostridia, including *Clostridium* species *C. asparagiforme*, *C. bolteae*, *C. scindens*, *C. indolis*, *C. ramosum*, and *C. hathewayi,* substantially increased the number of activated Tregs in the intestines of poly-colonized animals relative to GF and mono-colonized animals [51]. Furthermore, Tregs from GF mice exhibit reduced immunosuppressive function, as measured by IL-10 production, compared to *Clostridium*-colonized and conventional mice [50]. Thus, gut microbiota, including some strains of *Clostridium,* stimulate Tregs and protect against inflammation. Consistent with the finding that pro-inflammatory signaling from the immune system promotes tumorigenesis, ablation of Tregs in the setting of colitis and prior to tumor formation results in more severe colitis and the formation of a greater number of colon tumors in AOM/DSS-treated mice [52]. However, ablation of Tregs after tumor formation results in increased infiltration of the tumor by cytotoxic T cells, and a decrease in the number and size of the colon tumors. These data provide support for two opposing roles for Treg-induced immunosuppression during colon tumorigenesis: (1) protection against colonic inflammation and tumor initiation; and (2) a reduction in antitumor immunity after tumor initiation that promotes tumor growth.

## 3. Gut Microbiota Modulate the Response of Colon Tumors to Chemotherapy

### 3.1. Activation of Autophagy in Cancer Cells

In addition to directly modulating tumorigenesis through antitumorigenic and protumorigenic interactions with the colonic epithelium and underlying stroma, bacteria can also modify the response of tumors to therapy. For example, high levels of *F. nucleatum* in tumor tissues are associated with decreased recurrence-free [53] and overall survival [54] in CRC patients, indicating a potential role for *F. nucleatum* in modulating chemotherapy resistance. In vitro, coculture of CRC cell lines with *F. nucleatum* increased autophagy-related gene expression relative to culture in the absence of bacteria or coculture with other bacterial species, including *Prevotella intermedia*, *Parvimonas micra*, and *Peptostreptococcus anaerobius* [53]. Consequently, CRC cells cocultured with *F. nucleatum* exhibited reduced apoptosis in response to treatment with common CRC chemotherapies, such as 5-fluorouracil (5-FU) or oxaliplatin. Similar results were obtained in vivo: injection of xenograft tumors with *F. nucleatum* activated autophagy and in turn attenuated the antitumor activity of 5-FU or oxaliplatin. *F. nucleatum*-dependent chemotherapy resistance could be overcome by treatment of cells or mice with an autophagy inhibitor. Together, these data indicate that tumor-infiltrating *F. nucleatum* can activate autophagy and thus contribute to chemotherapy resistance in CRC. Interestingly, *F. nucleatum* levels predict response to adjuvant chemotherapy in esophageal squamous cell carcinoma, indicating a potential role for tumor-associated bacteria, such as *F. nucleatum*, in modulating the response of CRC, as well as extra-intestinal cancers to therapy [55].

### 3.2. Metabolism of Chemotherapeutic Agents

Metabolism of some therapeutic agents by specific gut-colonizing bacteria leads to structural alterations and changes in drug efficacy. In one study, 30 different chemotherapeutic agents were incubated for two hours in vitro with *E. coli* prior to filter-sterilization, and then added to cancer cell lines [18]. Preincubation of six agents, including 5-fluorocytosine and CB1954, with *E. coli* enhanced their cytotoxicity. Preincubation decreased the cytotoxicity of 10 other drugs, including Doxorubicin and Gemcitabine, as compared to those that were not preincubated with *E. coli*. Drugs with altered cytotoxicity exhibited changes on HPLC chromatograms indicative of biotransformation. The altered efficacy of some chemotherapeutic agents was further confirmed in vivo, using CT26 colon carcinoma isografts. Flank tumors were injected with *E. coli* or mock-colonized with sterile PBS, and chemotherapeutic efficacy was assessed by monitoring tumor volume over time, following intraperitoneal injection of either Gemcitabine or CB1954. Gemcitabine was less efficacious in decreasing the growth of tumors colonized with *E. coli*. Mice developed larger tumors and exhibited shorter survival times, as compared to mock-colonized mice treated in a similar manner. Conversely, the efficacy of CB1954 was enhanced in mice bearing tumors colonized with *E. coli*, leading to a decrease in tumor size and enhanced survival. When combined, these data indicate that common tumor-invasive bacteria, such as *E. coli*, can alter the biotransformation and efficacy of chemotherapeutic agents in vitro and in vivo. The potential impact of gut-colonizing bacteria on therapeutic efficacy may extend beyond the colon; the therapeutic response of lymphomas, melanomas, and lung carcinomas, as well as other tumor types, has been reported to correlate with changes in the composition of the gut microbiota [56].

### 3.3. Promotion of Antitumor Immunity

The gut microbiota can indirectly influence tumor response to therapy by modulating the antitumor immune response. Disruption of microbiota with broad-spectrum antibiotics impairs tumor response to CpG-oligonucleotide and anti-CTLA4 immunotherapies [19,20]. These therapies induce tumor cell death in mice bearing MC38 colon carcinoma isografts by promoting production of IL-17 and reactive oxygen species by tumor-infiltrating immune cells [19,20]. However, secretion of cytotoxic species by tumor-infiltrating immune cells was attenuated in mice treated with broad-spectrum antibiotics prior to immunotherapy. Consequently, mice treated with antibiotics and immunotherapies had larger tumors and shorter lifespans than mice treated with immunotherapy alone. Interestingly, the efficacy of anti-CTLA4 treatment in antibiotic-treated MC38-grafted mice could be rescued by colonizing mice with *B. fragilis*, immunizing with *B. fragilis* polysaccharides, or performing adoptive transfer with *B. fragilis*-specific T cells. Thus, activation of the immune system with microbial by-products is required for efficient tumor-cell killing in this model [20]. These effects are not limited to colon cancers; many genera of bacteria, including *Akkermansia*, *Bifidobacterium*, *Collinsella*, and *Enterococcus*, have been implicated in modulating response to immune checkpoint blockade in extra-intestinal cancers (e.g., melanoma, non-small cell lung cancer, and renal cell carcinoma) in both preclinical and clinical settings [57]. Thus, gut microbiota can modulate response to immunotherapy through activation of the immune system.

Given the critical role of the microbiota in shaping the maturation and activation of the immune system, it is likely that they also influence the efficacy of newly developed immunotherapeutics. Interestingly, antibiotic treatment prior to and following vaccination reduced vaccine-induced immune responses in humans, indicating that the microbiota may participate in vaccine-mediated immunity [58]. A recent surge of interest in stimulating antitumor immunity has resulted in the design of vaccines against antigens commonly expressed on CRC cells [59]. Vaccination of mice with antigens that are aberrantly expressed on colon tumor cells has been shown to prevent tumor formation [60], growth [61], and colonization in distant organs [62]. However, despite promising results in preclinical models, clinical trials to test the therapeutic efficacy of vaccines in CRC patients have yielded mixed results [59]. Future studies are needed to assess the similarity of the microbial and immune microenvironment of tumors in preclinical CRC models with that of human tumors, thus dictating the relevance of the observed efficacy of these vaccines in mice to a clinical setting.

## 4. Factors that Influence the Composition of the Gut Microbiota

Clearly, gut bacteria influence colon tumor formation, progression, and response to therapy in mouse models and humans alike, through interactions with the mucosal epithelium, metabolism of therapeutic compounds, and modulation of tissue-resident immune cells. Thus, variability in the colonic microflora of mouse models of CRC can influence disease penetrance, phenotypes, and experimental outcomes in these animals. These observations underscore the importance of understanding factors that influence the composition of the gut microbiota in mouse models.

### 4.1. Genetics

Genetic differences between mice may influence the resident gut microbiota [63,64,65]. Although genetic differences among strains likely influence the composition of the colonic microflora, specific strain influences are difficult to separate from strong environmental pressures, including cohort and litter effects. However, gene mutations and deletions that impact gut epithelial and/or immune homeostasis do appear to influence the resident gut microbiota. For example, when GF *Il10^−/−^* mice were acclimated to nonsterile conditions at weaning, the IL-10 deficient mice initially colonized (and maintained) a greater abundance of bacteria from the Enterobacteriaceae family, including *E. coli*, than wild-type mice [30]. Mutations in *Apc* may also drive divergent evolution of microbiota; six-week-old female C57BL/6J *Apc*^Min/+^ mice possess less diverse gut microbiota but greater relative abundance of *Bacteroidetes* spp than age- and gender-matched C57BL/6J wild-type mice [23]. However, animals in this study were born to dams in separate colonies and housed independently, providing an opportunity for natural drift of the colonizing microbiota in each strain. An evaluation of littermates (*Apc*^Min/+^ and wild-type) would be the best approach to deciphering the impact of mutations in genetic drivers of colon tumorigenesis on the gut microbiota.

### 4.2. Birth Mother

The composition of the colonic microbiota of a young mouse is initially dictated at or before birth by the mother [66,67]. Strain-specific differences in the composition of the gut microbiota largely disappear when embryos of multiple strains are implanted into a single mouse; instead, each pup develops the biome of the birth dam irrespective of strain. Thus, littermates tend to be colonized with highly analogous microbiota. Similarities are shared across generations; litters born to dams that are sisters are colonized with similar microbiota, whereas litters born to dams that are not sisters are colonized with divergent microbiota [68]. Importantly, colonization of pups by microbes from the dam can influence disease penetrance: C57BL/6 mice born from dams bearing conventional laboratory microbes developed more and larger tumors in response to AOM/DSS treatment than C57BL/6 mice born from dams bearing microbiota of wild-caught mice [17].

### 4.3. Age

The composition of gut microbiota changes rapidly in mice prior to weaning. Microbiota in young mice tend to be less diverse than that of older mice [69,70]. After initial colonization by vaginal microbes from the dam, the gut microbiota of pups shift, within the first few days of life, toward a low-diversity composition dominated by *Lactobacillus* [67]. As mice switch from nursing to solid food, the diversity of the gut microbiota increases rapidly to match the composition of the dam’s fecal material. After weaning, the microbiota equilibrates with that of co-housed animals, likely due to sharing of microbiota via ingestion of feces [65]. 

Aging leads to changes in the microbiota that can drive pro-inflammatory processes. Elderly mice (e.g., 18 months of age) exhibit increased systemic inflammation relative to young adult mice (2 months of age), characterized by increased serum levels of proinflammatory cytokines (IL-1β and TNFα) [71]. Age-induced changes in systemic inflammation correlate with decreased integrity of the gut barrier, and the increased relative abundance of specific genera of gut microbiota, including *Odoribacter*, *Butyricimonas*, *Gelria*, *Anaerosporobacter*, *Clostridium*, and *Oxalobacter*. The expression of pro-inflammatory genes was upregulated in the colonic mucosa of GF mice after colonization with microbiota from elderly mice vs. colonization with microbiota from young mice [72]. 

### 4.4. Housing

Gut microbiota are dynamic, and changes occur naturally over time. Even mice that start with highly similar and defined gut microbiota, including germ-free mice that are simultaneously acclimated to a nonsterile environment or gavaged with specific bacteria, develop divergent gut microbiota over time [69,70,73]. In one study, GF mice were inoculated with a defined microbiome and then housed either in microisolators or in individual ventilated cages [69]. The microbiota that developed in mice housed in microisolator cages differed from that of mice housed in individual ventilated cages. Irrespective of the type of cage, taxa were identified over time that were not present in the original inoculum. Furthermore, the number of genera detected increased significantly within three months, demonstrating that the composition of the gut microbiota among animals in a single cage can drift rapidly away from that of the original inoculum. Consistent with this observation, animals housed in the same cage exhibit significantly less inter-individual variation in the composition of the gut microbiota than animals housed in independent cages [65]. This natural microbial drift may contribute to variability in disease phenotype and experimental outcomes between cages. For example, the degree of inflammation observed in mice treated with a colitis-inducing agent varies significantly more among mice housed in different cages than in mice housed together in the same cage [70]. 

### 4.5. Diet

Diet heavily influences the composition of gut microbiota in the adult host. Shifts in the composition of the microbiota of laboratory mice can be detected within 48 hours following a dietary modification [74]. These shifts may influence disease penetrance. Mice fed a diet high in the milk protein casein harbor gut microbiota with less diversity, characterized in part by decreased relative abundance of Firmicutes and increased Bacteroidetes, and develop more severe DSS-induced colitis than mice fed a diet low in casein. A diet high in psyllium, a soluble plant fiber, increased microbial diversity and decreased severity of DSS-induced colitis in mice compared to a diet high the insoluble fiber cellulose. The aggravating effects of casein protein and protective effects of soluble fiber on DSS-induced colitis are attenuated in GF mice relative to conventional mice, indicating that gut microbiota are responsible in part for modulating disease severity.

The way in which the rodent chow and water are sterilized prior to administration influences the composition of the gut microbiota in recipient mice. The number of bacterial species in the gut of mice maintained on irradiated chow is lower than that of mice maintained on autoclaved or untreated chow [75]. In addition, the relative abundance of microbial phyla, including Firmicutes and Bacteroidetes, are altered. Mice given autoclaved water, as compared to that sterilized by H_2_SO_4_ acidification, exhibit a reduction in microbial diversity and a change in the abundance of various microbial taxa [76]. Interestingly, NOD mice maintained on acidified drinking water develop Type 1 Diabetes (T1D) more rapidly than NOD mice maintained on neutral pH drinking water [77]. The change in incidence and rate of developing T1D is preceded by differences in the number and relative abundance of specific genera of gut microbes, including a decreased prevalence of *Bacteroides.* In addition, *Parabacteroides* and *Prevotella* are acquired when mice are switched from acidified water to neutral pH water. Thus, the ability of the gut microbiota to impact disease susceptibility in mouse models is influenced by the nutrient composition of the diet, and the manner in which the diet and drinking water are sterilized.

### 4.6. Institution

The influence of environmental factors on the composition of the gut microbiota is compounded over generations, and thus identical strains of mice housed at different institutions can exhibit dramatic differences in the composition of their gut-resident microflora. Examination of the gut microbiota of C57BL/6J breeding stocks from 21 different animal facilities revealed profound differences [75]. Variability in animal housing, handling, and care likely contributed to this heterogeneity; treatment of the chow (untreated, irradiated, or autoclaved), type of housing (whether using individually ventilated cages or not), the vendor who supplied the mice, and the presence of other mouse strains in the facility all influenced the number of bacterial species identified and their relative abundance. 

Importantly, differences in the composition of the microbiota at different institutions has been shown to influence disease susceptibility when utilizing the same mouse model [78]. *Il10^−/−^* mice serve as a prototypic example of the impact of institutional-specific microbiota on disease severity. The investigators who initially developed the *Il10*^−/−^ mouse observed that mice exhibited severe enterocolitis, resulting in anemia, weight loss, and mortality by 4–12 weeks of age. Colitis was attenuated in mice housed under specific pathogen-free (SPF) conditions relative to those housed under conventional conditions [79]. Subsequent studies revealed that SPF *Il10*^−/−^ mice housed at some institutions readily developed extensive colitis, while *Il10^−/−^* mice housed at other institutions fail to develop colitis [11,12,13]. Furthermore, *Il10^−/−^* mice infected with *H. hepaticus* developed more severe colitis than uninfected mice at one institution but not at another; GF mice never developed colitis [12,13]. Thus, the penetrance and severity of disease phenotypes in mouse models can change dramatically when mice are rederived at new institutions, likely due to colonization by different microbiota.

### 4.7. Immune System

The composition of the gut microbiota is influenced significantly by the immune system of the mouse. Mice that lack functional adaptive immune systems harbor biomes with a modified composition relative to wild-type mice [80,81,82]. For example, the intestinal microbiota of immunodeficient C57BL/6J *Rag1*^−/−^ mice that lack mature lymphocytes contain taxa that are significantly different from those of C57BL/6J wild-type (*Rag1*^+/+^) mice, including decreased relative abundance of Lactobacillales and increased species of Verrucomicrobiales, such as *Akkermansia muciniphila* [80]. However, the abundance of *A. muciniphila* in *Rag1*^−/−^ mice bearing bone marrow grafted from *Rag1*^+/+^ mice was similar to that of *Rag1*^+/+^ mice, indicating that the adaptive immune system may play a role in modulating the abundance of some microbial species within the gut. Inflammation produced as a consequence of immune dysregulation likely also influences gut microbial communities. This may explain the shifts in microbiota observed in mice after DSS treatment, as well as shifts in the gut communities of *Il10*^−/−^ vs. wild type littermates [15,30]. These data serve as evidence of the ability of the immune system to shape the gut microbial communities.

## 5. Implications for Model Selection and Experimental Design

Gut microbiota coexist in a delicate balance with the colonic mucosa and have the power to either restrain or promote colon cancer. Unfortunately, factors that influence the microbiota can have unexpected consequences in preclinical tumor models, negatively influencing experimental reproducibility and the broad applicability of findings. Phenotypes initially attributed to specific gene mutations or mouse strains may instead arise as a result of litter or cage effects [63,64]. Mouse models that readily develop colonic inflammation at one institution can remain totally healthy at another center [12,13]. Therapies that appear effective in specific mouse models may lose their potency or have unexpected side effects in other models and/or humans due to differences in gut microbiota and immunity [18,83,84]. Through in-depth characterization of the model to be used and with significant attention to experimental design and systematic reporting, researchers can circumvent major sources of variability that hinder translatability, thus ensuring that their data are accurate and can be used to advance the efforts to prevent and treat colon cancer. 

### 5.1. Characterizing Microbiota to Improve Mouse Models of CRC

As discussed above, variations in the gut microbiota of laboratory mice can influence disease phenotypes and experimental outcomes in mouse models of CRC. Characterization of the gut microflora throughout an experiment allows investigators to do the following: (1) quantify the number and abundance of microbial genera present in the model; (2) determine how the microbiota change over the course of the experiment; and (3) understand how the microbiota interact with the colonic mucosa to influence disease phenotypes in the model. Characterization is routinely accomplished through 16s rRNA gene sequencing, which can identify and quantify the microbial genera present within the gut [85]. Using this approach, the number and identity of each unique genus, as well as its abundance among the total microflora, can be assessed. Importantly, the methods used to collect, store, and analyze microbiota from colon samples can influence the sequencing results [86,87]. For example, sequencing results can vary significantly depending upon which regions of the 16s gene are selected for analysis [87]. Furthermore, the sequencing platform selected can yield different results; the Illumina MiSeq and Ion Torrent PGM sequencing platforms differ in their sensitivity to detect specific microbial species and assessment of overall diversity, as indicated by scores calculated from sequencing data [86]. As an alternative to 16s rRNA targeted sequencing, shotgun whole genome sequencing (WGS) is sometimes used [85]. This approach provides additional information about the gut-resident microbiota, as it allows detection of genes required for the production of toxins and other key metabolites that can influence microbial activity within the gut. Notably, WGS detects more total species, identifies different relative abundances, and yields higher diversity scores than 16s rRNA sequencing of the same samples [88]. Given the dramatic impact that gut-microbiome-sequencing methods can have upon the results obtained, standardization and clear reporting of procedures used throughout an experiment are critical for accurate interpretation and direct comparison of results to those obtained by others.

While knowledge of the overall composition of the gut microbiome represents a step toward greater understanding of how the microflora influence disease phenotypes in mouse models, it provides limited information about the functional activity of the microbes. Emerging technologies, including transcriptomics, proteomics, and metabolomics, allow investigators to examine the activity of members of the resident microbiota [85]. These data should enhance our understanding of how the microbiota interact with the colonic mucosa to either restrain or promote tumorigenesis, and may ultimately inform strategies to improve therapeutic interventions. Furthermore, these data are anticipated to facilitate the more accurate interpretation of variable experimental results obtained from preclinical CRC models.

### 5.2. Modifying Microbiota to Improve Mouse Models of CRC

Some institutions have tried to standardize the gut microflora of laboratory mice via rederivation using dams colonized with defined microbial inoculum, such as the altered Schaedler flora [75]. Although this results in initial colonization with defined species, it does not prevent rapid drift from the composition of the initial inoculum, based on institutional- and cage-specific environmental factors, such as those described above [65,69,75]. Many institutions maintain their mouse colonies under specific pathogen-free (SPF) conditions; however, ‘SPF status’ provides no information about the composition of the microbiota and does not imply similarity to SPF mice at other institutions. SPF mice are tested regularly to ensure they remain free of a set of predefined pathogens. Since each institution defines its own criteria for acceptable pathogens and SPF status, the gut microbiota of SPF mice at different institutions and from different vendors can vary dramatically both in the types and relative abundance of microbial species present [75,89]. 

Manipulation of the gut microbiota may be required to establish mouse models that more accurately mimic human immune responses throughout tumorigenesis and dictate response to therapy. While mouse strains have been used to test immunotherapies alone or in combination with conventional chemotherapeutic agents [60,61,62,90,91], these models are usually selected without considering whether the tumor immune microenvironment recapitulates that of human cancer patients. In any event, the microenvironment of the tumor clearly impacts the response of the tumor to therapeutic interventions [18,83]. Given the important role of the microbiota in the tumor microenvironment, through direct interactions with both the tumor and resident immune cells, one approach to mimicking the tumor microenvironment in mice may be to ‘humanize’ the murine gut with microbiota from the human gut. Unfortunately, microbial species from humans colonize mice with variable efficiency, resulting in a bias for certain species (especially members of the *Bacteroides* and *Parabacteroides* genera) over others [92,93]. In addition, immune infiltration and activation within the gut of mice bearing human-derived microbiota is less mature than that within the gut of mice bearing conventional microbiota [44]. Additional research is required to determine whether mice bearing ‘humanized’ gut microflora accurately model various aspects of human disease progression, including activation of the immune system and response to therapy.

Alternatively, embracing the natural microflora of the mouse may lead to models that better recapitulate human immune responses. The diversity of gut microbiota in wild mice is significantly higher than that of conventional laboratory mice [84]. Rederivation of laboratory mice by transplantation of C57BL/6 embryos into pathogen-free, wild-caught pseudopregnant *Mus musculus domesticus* results in ‘wildling’ C57BL/6 mice colonized with the diverse microbiota of wild mice. Although genetically identical to conventional C57BL/6 mice, the immune cell landscape of the gut of most wildlings differed from that of conventional mice, including increased numbers of cytotoxic T cells and decreased numbers of NK cells. Interestingly, immune responses of wildling mice more closely mimicked human immune responses to CD28-superagonist therapy and TNF-α blockade than that of conventional laboratory mice. Additional research is required to determine whether wildling mice also phenocopy human immune responses during therapeutic treatment of colon tumors.

### 5.3. Experimental Design—Correcting for Factors that Influence Gut Microbiota in Experiments

While some factors that influence the colonic microflora can be controlled, such as sterilization of drinking water, other factors, including natural drift within colonies over time, are more elusive. Given the staggering number of subtle environmental factors that can influence gut microbial diversity and composition, many of which are beyond the control of the investigator, it is impossible to perfectly standardize and replicate all conditions performed within a single laboratory, much less across all laboratories worldwide. In fact, controlling and standardizing all variables that influence microbial composition and phenotypic outcomes within mouse models of CRC is not desirable. Lack of heterogeneity between mice, as seen in humans, may contribute to biases in study results. Such findings may be highly specific to the conditions of the experiment instead of broadly applicable and translatable to the inherent heterogeneity of humans [94,95,96]. Instead, some environmental variability between subjects within an experiment may be ideal, providing it is applied equally across all groups. Heterogeneity can be introduced quite naturally into an experiment by enrolling mice in batches over time, thus ensuring that subtle changes in the environment are introduced over the course of the experiment. Batch-specific effects can then be estimated and accounted for through appropriate statistical modeling approaches. 

To correct for factors that strongly influence the microbiota, appropriate randomization of mice to study groups is essential. Block randomization, based on factors that are likely to confound experimental outcomes (e.g., litter), ensures that confounding variables are equally spread across all treatment groups. Housing of animals should be carefully considered and recorded. Co-housing mice from multiple experimental groups will help standardize the microbiota across animals in each group. However, if the outcome of the experiment is dependent in part on the gut microbes, co-housing animals may make it difficult to detect the effect, as mice will participate in coprophagy and thus share microbiota. Regardless of the approach employed, it is important to provide these details so other investigators can understand and replicate the experiments. 

### 5.4. Reporting Experimental Details

In an effort to improve reproducibility in animal research, Kilkenny et al. published a set of comprehensive guidelines for reporting animal research [97]. The ARRIVE guidelines encourage researchers to report strain and environmental factors that might influence the composition of the gut microbiota and immune system in experimental models, including providing international strain nomenclature, genotype, age, sex, and weight, as well as the husbandry conditions, housing type, diet, time of day the experiment was performed, and the pathogen status of the experimental animals. While it is impossible to control and replicate all environmental conditions that contribute to diversity of the gut microbiota and immune system, cataloguing and reporting these factors make it easier for other researchers to replicate studies precisely and identify the basis for results that differ. Thus, thorough and systematic reporting of study variables by following the ARRIVE guidelines could improve replicability and lead to the discovery of unexpected interactions between environmental, microbiological, immunological factors and cancer biology.

New insights into colon tumor biology are continuously arising from the careful study of both well-established and newly designed mouse models. Clearly, interactions between the gut microbiota and the host immune system influence colon-tumor outcomes. Through careful characterization of mouse models, appropriate study design, and clear reporting of environmental and experimental conditions, mouse models can continue to rapidly advance our understanding of the complex biology that contributes to colon cancer initiation, progression, and therapeutic response.

## Figures and Tables

**Table 1:** Influence of microbiota on disease phenotype in common mouse models of CRC (colorectal cancer).

Tumor Induction	Mouse Model	Impact of Microbiota on Colon Phenotype	References
Sporadic Familial Adenomatous Polyposis	*Apc* ^Min/+^ *Apc* ^MinΔ716/+^ *Cdx2-Cre Apc* ^flox/+^	Mice administered continuous broad-spectrum antibiotics develop fewer colon tumors, whereas mice administered intermittent antibiotics develop more tumors. Infection of *Apc*^Min/+^ mice by *Fusobacterium nucleatum,* or *Apc*^Min716/+^ mice by enterotoxigenic *Bacteroides fragilis* and/or *pks+ Escherichia coli* increases tumor multiplicity.	[5,6,7,8,9]
Inflammation	*Il10* ^−/−^	Germ-free mice do not develop intestinal inflammation. Differences in the composition of microbiota influence severity of sporadic colitis in mice housed at different institutions. Infection with *E. coli* increases tumorigenesis following azoxymethane (AOM) treatment.	[10,11,12,13]
DNA mismatch repair deficiency	*Msh2* ^−/−^	Germ-free and antibiotic (broad-spectrum)-treated *Apc*^Min/+^ *Msh2*^−/−^ mice develop fewer colon tumors than ‘conventional’ untreated mice (bearing natural microbiota).	[14]
Chemical induction	AOM/DSS	Treatment with either AOM or dextran sodium sulfate (DSS) changes the composition of the gut microbiota. Germ-free mice exhibit delayed tissue repair and develop more tumors than conventional mice. Conventional C57BL/6 mice develop more tumors than the genetically identical mice colonized with microbiota from wild-caught mice.	[15,16,17]
Transplantation	CT26 MC38	*E. coli* modifies the response of tumors to chemotherapy. Depletion of microbiota by broad-spectrum antibiotics attenuates the response of tumors to immunotherapeutics.	[18,19,20]
